# Recurrent Ileal Conduit Obstruction Secondary to Parastomal Hernia Resulting in Bilateral Hydronephrosis and Pyelonephritis

**DOI:** 10.7759/cureus.114398

**Published:** 2026-08-12

**Authors:** Yogendran Kandasamy, Muhammad Ashif Khan Abdullah, Ahmed Galal, Shobhana Murugesan, Jarin Tuli

**Affiliations:** 1 Urology, Countess of Chester Hospital NHS Foundation Trust, Chester, GBR; 2 Emergency Medicine, Craigavon Area Hospital, Craigavon, GBR; 3 Geriatrics and Stroke, Royal Liverpool and Broadgreen University Hospitals NHS Trust, Liverpool, GBR

**Keywords:** extrinsic compression of the ileal conduit, ileal conduit urinary diversion, intraconduit catheterization, obstructed urinary system, obstructive hydronephrosis, parastomal hernias, postobstructive aki

## Abstract

Parastomal hernia is a recognized long-term complication following ileal conduit urinary diversion, though associated urinary obstruction is not very common. We report the case of a 63-year-old woman with an extensive history of abdominal and urological surgery who presented with bowel obstruction symptoms, reduced conduit output, and acute kidney injury. Cross-sectional imaging demonstrated bilateral hydronephrosis and hydroureter along with bilateral pyelonephritis secondary to extrinsic compression of the ileal conduit by a parastomal hernia containing the cecum. Intraconduit catheterization made a significant clinical and biochemical recovery through decompression. In cases of prohibitive operative risk, long-term conduit catheterization became the practice based on multidisciplinary agreement. The case has brought to focus the diagnostic and management issues of rare urinary complications due to parastomal hernia in patients with hostile abdomens.

## Introduction

An ileal conduit is the most commonly performed urinary diversion following radical cystectomy, in which a short segment of ileum is used to create a conduit that carries urine from the ureters to a urostomy on the abdominal wall [1,2]. Long-term complications include stomal stenosis, infection, and parastomal hernia [3].

A parastomal hernia is a common long-term complication of stoma formation, characterized by protrusion of abdominal contents through the abdominal wall defect adjacent to the stoma, with reported incidence rates ranging from 17% to over 50% [4,5]. Although parastomal hernias are often asymptomatic, they can sometimes lead to bowel obstruction or weakening of stoma function [1,4]. More commonly, upper tract dilatation in ileal conduit patients is related to stomal stenosis, ureteroenteric strictures, or intrinsic conduit pathology rather than extrinsic compression [2,6].

As the parastomal hernia enlarges, the herniated contents occupy the confined space surrounding the ileal conduit, which exerts pressure on the conduit from the outside, particularly at the abdominal wall, causing mass effect and collapsing the ileal conduit [7]. This extrinsic compression of an ileal conduit impairs urinary drainage, resulting in urinary stasis and progressive back pressure to the upper urinary tract, leading to hydroureter, hydronephrosis, and postrenal acute kidney injury (obstructive uropathy), which is rare as in this case report [8,9]. This is especially complicated in this scenario with multiple prior laparotomies, for which surgical reintervention has high morbidity and is beyond consideration [10].

## Case presentation

A 63-year-old woman presented with a five-day history of abdominal pain, associated with nausea and vomiting, absolute constipation, and markedly reduced urine output from her ileal conduit. She has a complex surgical background due to severe interstitial cystitis requiring multiple major abdominal and urological procedures, including clam cystoplasty, revision of clam cystoplasty, cystectomy, and urethrectomy with ileal conduit formation, emergency repair of parastomal hernia for small bowel obstruction, and revision of the ileal conduit following stomal wall necrosis. She had also experienced recurrent small bowel obstructions, which were managed conservatively. On presentation, she was systemically ill with fever, pain, and tenderness of the abdomen in the region of the stoma with reduced urine output. Laboratory investigations demonstrated markedly elevated inflammatory markers and acute kidney injury stage 3.

Imaging

**Figure 1:**
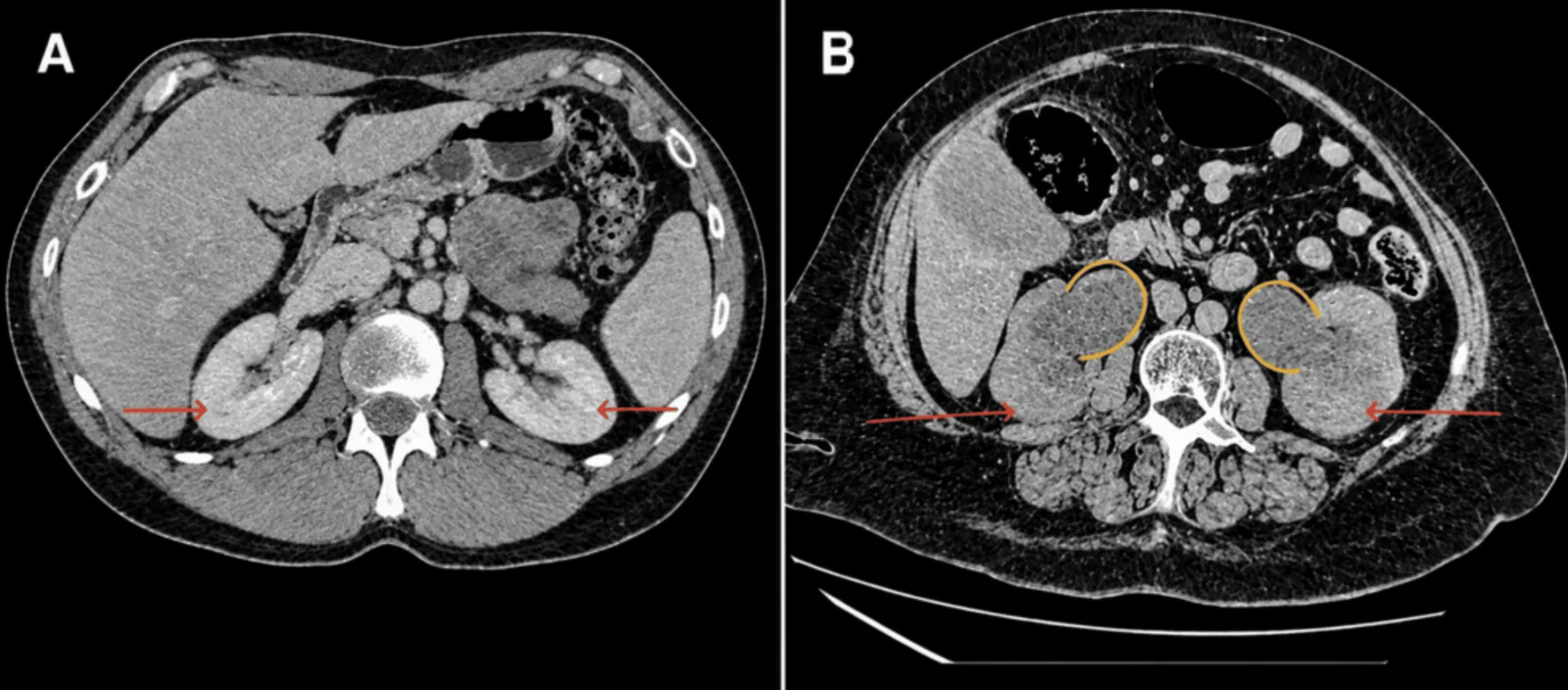
Axial view of the CT abdomen (A) Normal kidneys (red arrows) without hydronephrosis. (B) Bilateral moderate hydronephrosis (yellow line) and kidneys (red arrows) CT: computed tomography

**Figure 2:**
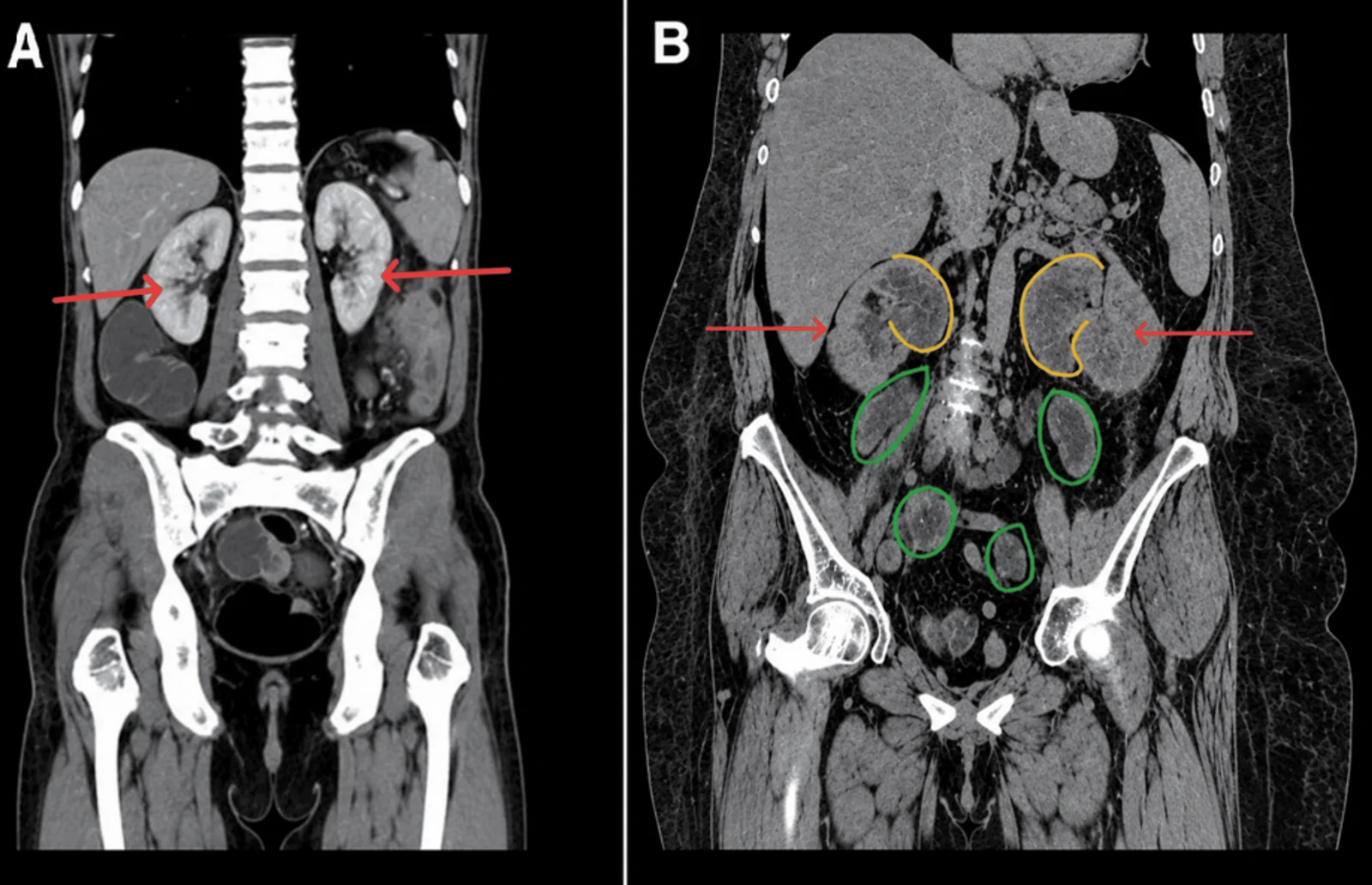
Coronal view of CT abdomen (A) Normal kidneys (red arrows) without hydronephrosis. (B) Bilateral moderate hydronephrosis (yellow line), bilateral hydroureter (green circles), and kidneys (red arrows) CT: computed tomography

**Figure 3:**
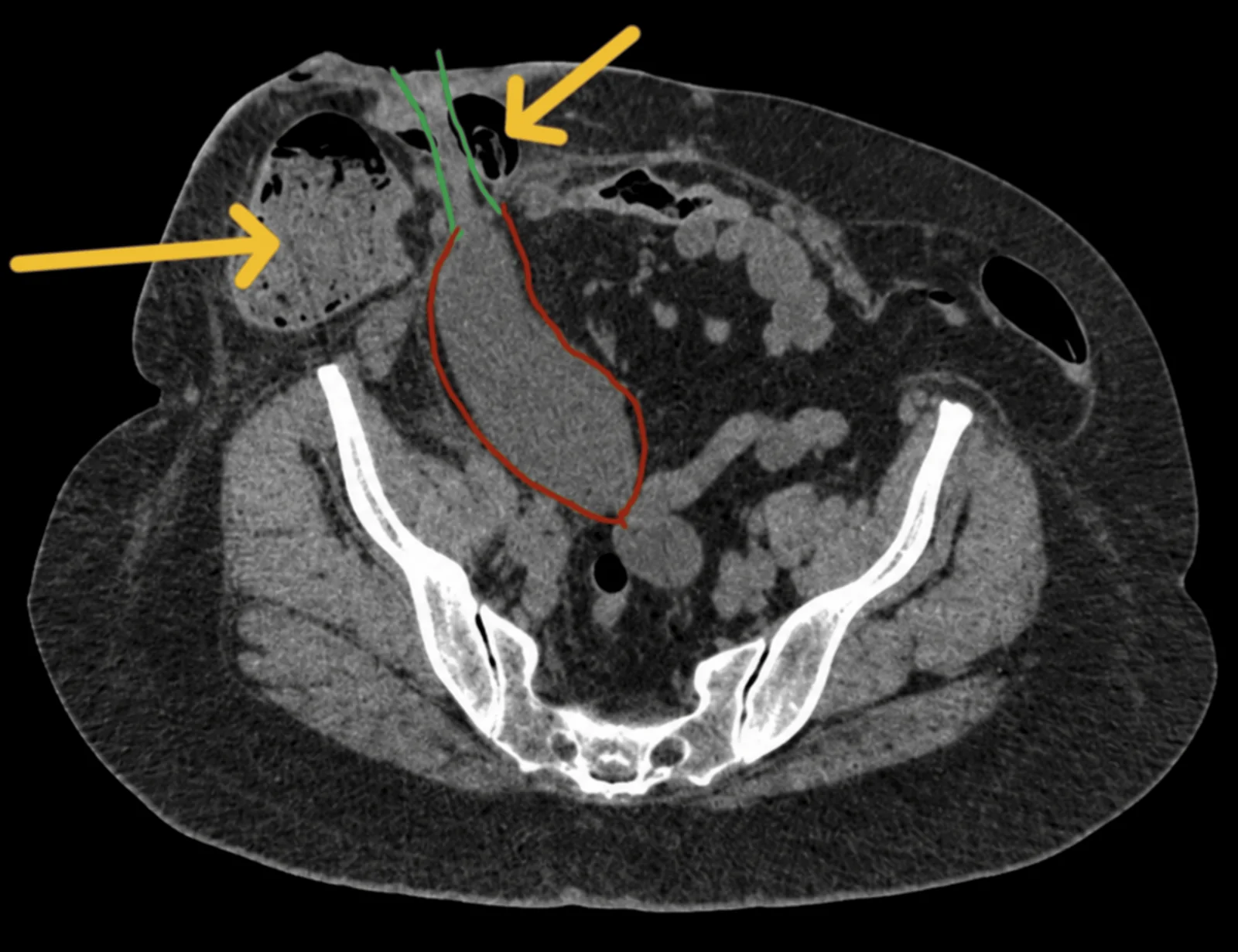
Axial view of CT abdomen The narrow tubular structure between the green lines is the distal segment of the ileal conduit being compressed by the large parastomal hernia shown by the yellow arrows on both sides of the conduit. The structure represented by the red line is the dilated proximal segment of the ileal conduit secondary to mechanical obstruction CT: computed tomography

Computed tomography (CT) of the abdomen and pelvis with contrast demonstrated moderate bilateral hydronephrosis and hydroureter with peripheral fat stranding, as shown in Figures 1, 2. Figure 3 demonstrates distension of the intra-abdominal portion of the ileal conduit and a parastomal hernia containing the cecum, causing significant extrinsic compression of the conduit at the abdominal wall, resulting in mechanical obstruction.

Management

**Figure 4:**
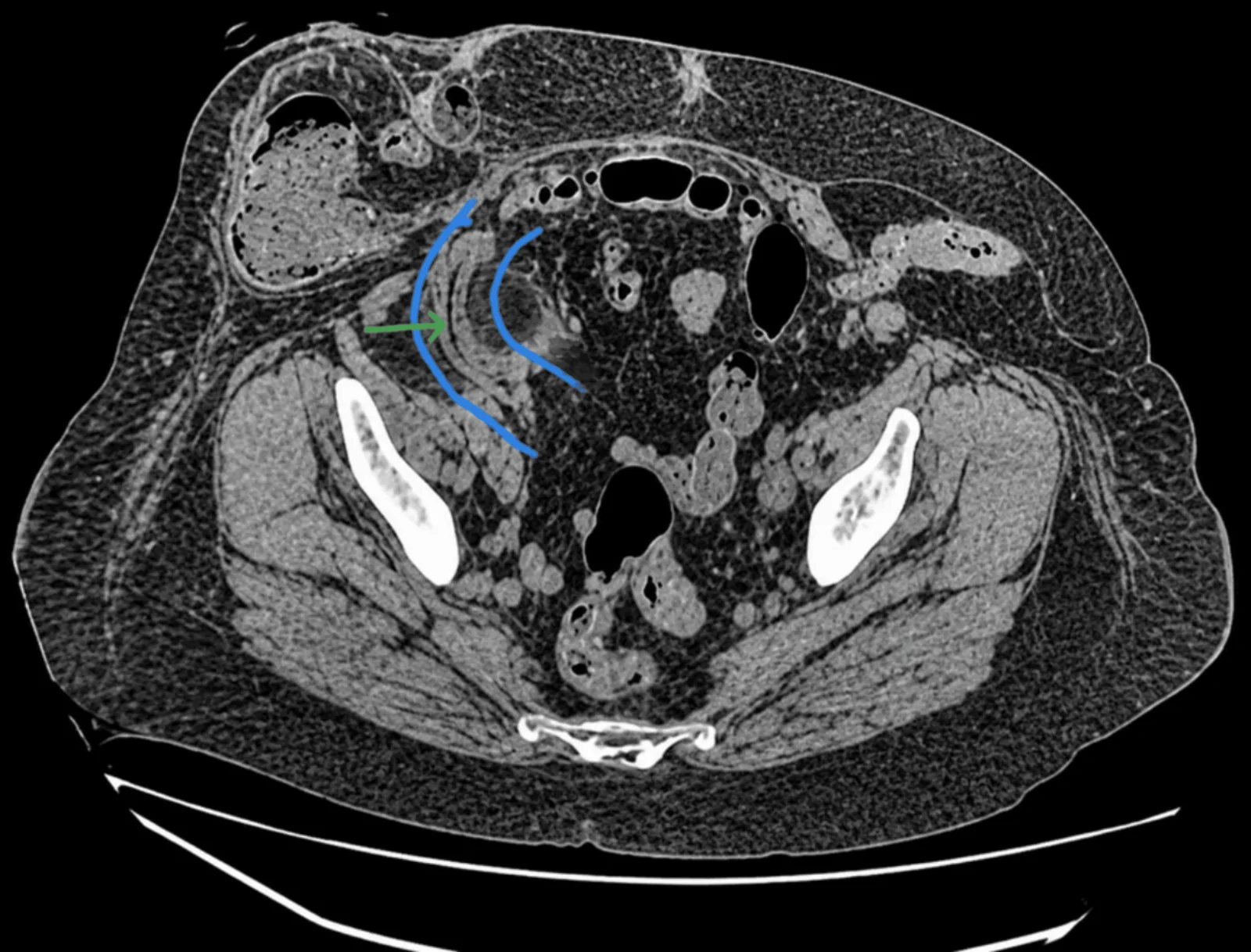
Axial view of the CT abdomen The image shows a collapsed ileal conduit outlined by blue lines, and the green arrow indicates the catheter within the conduit CT: computed tomography

**Figure 5:**
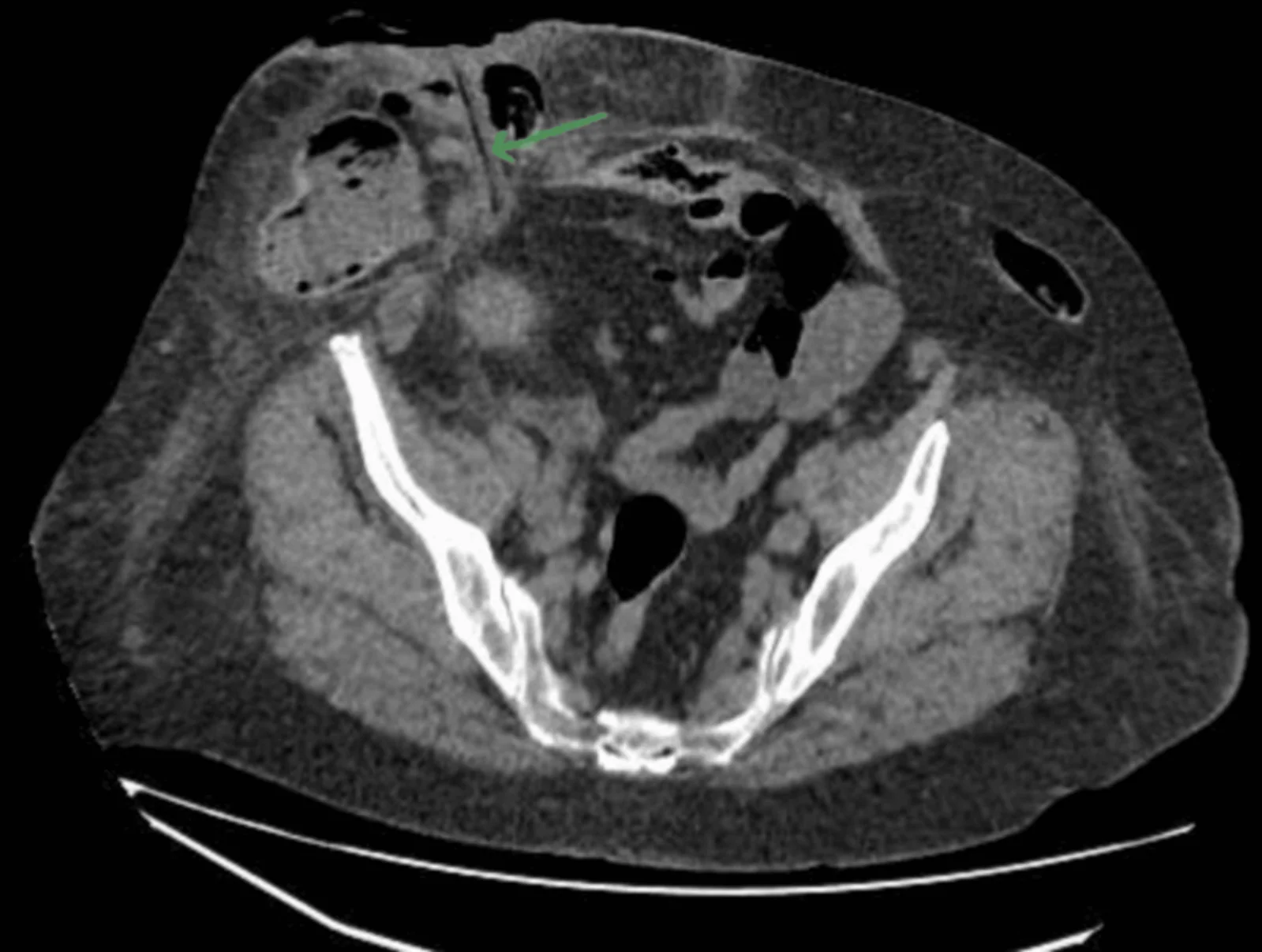
Axial view of CT abdomen The image shows a catheter (green arrow) within the ileal conduit in the anterior abdominal wall CT: computed tomography

A 16-Fr Foley catheter was inserted into the ileal conduit via the urostomy (Figures 4, 5) using a sterile technique, following the same principles as standard urethral catheterization. Instillagel was used as a lubricant to facilitate atraumatic insertion. The catheter balloon was deliberately left deflated to avoid potential complications, including mucosal injury, pressure erosion, and ischemic damage to the ileal conduit. The catheter insertion helped drain urine output from the ileal conduit proximal to the part compressed by the parastomal hernia, thereby bypassing the mechanical obstruction.

Immediately following catheter insertion, 420 mL of urine was drained within the first 15 minutes. Urine output subsequently totaled 1,150 mL within the first four hours and reached 5,350 mL over the initial 24-hour period. This marked increase in urine output was consistent with postobstructive diuresis following relief of obstructive uropathy. The patient experienced rapid symptomatic improvement with resolution of abdominal pain and progressive normalization of renal function and inflammatory markers within 48 hours of presentation.

**Figure 6:**
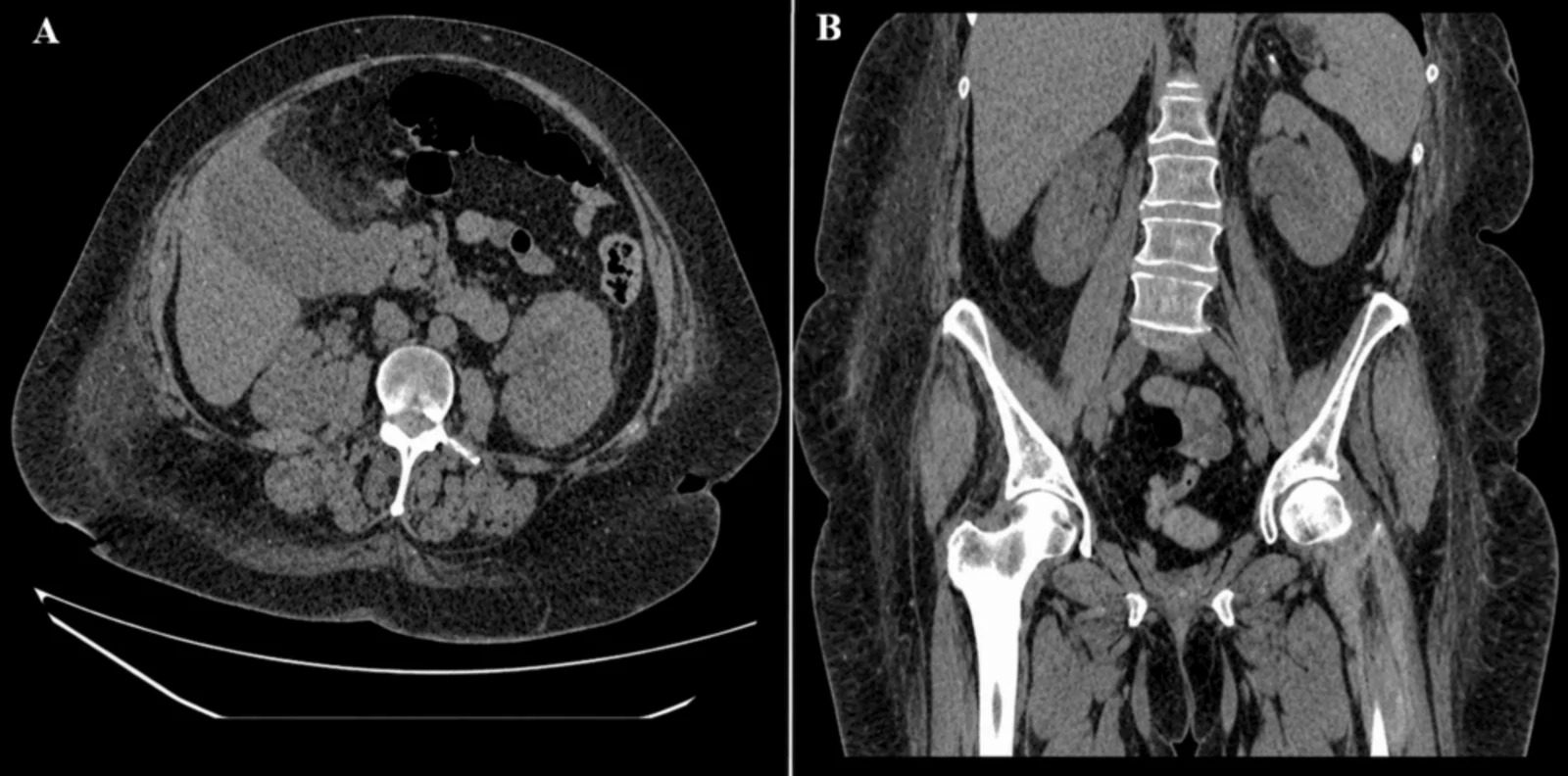
(A) Axial and (B) coronal views of CT abdomen The image shows the resolution of hydronephrosis post-catheter insertion CT: computed tomography

CT confirmed decompression of the ileal conduit and improvement in bilateral hydronephrosis (Figure 6). Interventional radiology determined that obstruction was distal to the ureteroileal anastomoses; therefore, percutaneous nephrostomy was not required. Conduit catheterization was deemed the most appropriate decompressive strategy [9].

Given extensive prior surgeries and hostile abdominal anatomy, further parastomal hernia repair was considered prohibitively high risk. Surgical repair of parastomal hernia carries significant recurrence rates and morbidity, especially in reoperative abdomens [1,8].

Complications

During admission, two episodes of catheter dislodgement led to recurrent abdominal pain and decreased conduit output. Repeat imaging confirmed recurrence of bilateral hydronephrosis, more pronounced on the right side. Reinsertion of the catheter restored drainage. The catheter balloon was therefore inflated within the conduit to prevent migration. The balloon was only inflated with 5 mL of distilled water, as the risk of complications mentioned above was managed. The catheter did not get displaced further after fixation until the next catheter change.

Outcome and follow-up

Following secure catheter placement, renal function improved markedly to her baseline within 48 hours, with estimated glomerular filtration rate increasing from 11 to 70 mL/minute/1.73 m² and creatinine improving from 575 to 140 µmol/L. Her white cell count was 18.5, which normalized within three days of intravenous antibiotics. Pain resolved quickly after catheter insertion.

A multidisciplinary decision was made for long-term ileal conduit catheterization with periodic changes every 12 weeks under urological supervision in hospital, as district nurses might find it challenging to change in the community.

No further issues were encountered, such as catheter dislodgement and obstructive uropathy, when the patient came for the next two catheter changes in the Urology unit.

## Discussion

Parastomal hernia remains one of the most common late complications of ileal conduit diversion [1,2,8]. Although often asymptomatic, it may lead to bowel obstruction or stomal dysfunction [1,8]. Urinary obstruction due to external compression is rare.

Upper tract dilatation in ileal conduit patients most commonly results from ureteric strictures or stomal stenosis [3,4]. Only limited case reports describe bilateral hydronephrosis caused by parastomal hernia [5,6]. The mechanism involves extrinsic compression of the conduit at the abdominal wall, resulting in distal obstruction.

Prompt recognition is essential. Reduced conduit output should prompt urgent imaging to evaluate for mechanical obstruction [7]. CT imaging remains the modality of choice.

While surgical repair may be appropriate in selected patients, recurrence rates and morbidity are considerable, especially in those with complex surgical histories [1,8]. In high-risk patients, conduit catheterization may serve both diagnostic and therapeutic purposes [9].

This case highlights the importance of considering extrinsic compression as a cause of obstructive uropathy in patients with ileal conduits and demonstrates that long-term catheterization can provide effective and durable decompression when surgery is contraindicated.

## Conclusions

Parastomal hernia can rarely cause extrinsic compression of an ileal conduit, resulting in bilateral hydronephrosis, pyelonephritis, and acute kidney injury. Long-term conduit catheterization could be considered as an alternate safe and effective management approach for patients in whom surgical reintervention has high morbidity.
